# Entomopathogenic Nematode *Steinernema carpocapsae* Venom Proteins Disrupt Developmental Physiology and Reproduction of *Spodoptera frugiperda* (Lepidoptera: Noctuidae)

**DOI:** 10.3390/toxins18040185

**Published:** 2026-04-14

**Authors:** Manisha Mishra, Leonor Georgette Farias, Steven Song, Steven Nguyen, Purav Shah, Adler R. Dillman

**Affiliations:** Department of Nematology, University of California, Riverside, CA 92521, USA; manisham@ucr.edu (M.M.); purav.shah@email.ucr.edu (P.S.)

**Keywords:** *Steinernema carpocapsae*, *Spodoptera frugiperda*, entomopathogenic nematodes, venom proteins, biopesticides

## Abstract

The use of *Steinernema carpocapsae* infective juveniles as biological control agents is a long-standing practice, yet the oral impact of their secreted venom proteins on crop pests remains largely unknown. We evaluated the oral toxicity of *S. carpocapsae* venom proteins against *Spodoptera frugiperda* using artificial diet assays. Ingestion caused significant dose-dependent toxicity in early-instar larvae, resulting in mortality and a prolonged developmental duration. Carry-over effects were profound; treated pupae were smaller and malformed, with only 19% of larvae fed on 1000 ng g^−1^ venom protein-supplemented diet reaching adulthood compared to 92% in controls. Surviving adults lived 30% fewer days and laid over 90% fewer morphologically normal eggs. These physiological disruptions coincided with elevated oxidative stress and detoxification enzyme activity, suggesting the venom induces oxidative and detoxification responses, which may be associated with the observed phenotypic alterations. This study provides the first demonstration of the oral toxicity of entomopathogenic nematode venom proteins, positioning them as a promising resource for the discovery of novel insecticidal proteins for sustainable pest management.

## 1. Introduction

With the increasing demand for chemical-free crop produce, the usefulness of other insect control measures has tremendously increased, such as the potential use of entomopathogenic nematodes (EPNs) as biological control agents. EPNs are obligate parasites of insects. They are highly efficient and rapid in seeking and killing hosts [1]. The pathogenic properties of parasitic nematodes are largely attributed to their excretory/secretory (ES) products, which we refer to as venom [2]. However, their practical application in sustainable agriculture is limited by challenges such as their short shelf life and sensitivity to sunlight and UV radiation [3].

Entomopathogenic nematode venom is largely a mix of pathogenic proteins [2,4]. These proteins are released during infection and play a critical role in host tissue degradation and immune suppression [4,5]. The broad-spectrum toxicity of EPN venom proteins against various insects has been previously established when injected subcutaneously [4,6]. A substantial body of literature has characterized the biological activity of individual venom proteins from *S. carpocapsae*, including diverse serine proteases, metalloproteases, aspartic proteases, and protease inhibitors, demonstrating their ability to degrade host tissues and actively modulate insect immune responses such as the melanization cascade, hemocyte aggregation, and clot formation [7,8,9,10,11,12,13,14,15,16,17]. Building on this foundational work, recent secretomic profiling revealed that activated *S. carpocapsae* release a highly complex, lethal venom cocktail comprising hundreds of proteins [4]. Specific effectors within this venom, such as fatty acid- and retinol-binding proteins (FARs) and secreted phospholipases (sPLA2), have been shown to profoundly compromise host cellular and humoral immunity [18,19].

While previous studies have elegantly demonstrated the in vivo toxicity of these proteins, they have primarily relied on direct delivery into the insect hemocoel, either through injection or transgenic expression [4,18,20], effectively bypassing the digestive tract. Therefore, the consequences of oral exposure to these venom proteins have received far less attention and remain largely unexplored, particularly with respect to their impact on crop pests. Although one study investigated the oral exposure of an *S. carpocapsae*-derived fusion ScK1 protein, showing pronounced sublethal toxicity and significantly reduced locomotor activity in fruit flies [20]. This prior work was limited to a single, isolated, recombinantly expressed protein rather than the complete, naturally secreted venom. Consequently, the oral toxicity of the entire *S. carpocapsae* venom cocktail remains largely unexplored. This represents a critical knowledge gap that we have sought to address.

Systematic evaluation of these venom proteins under dietary exposure conditions is therefore indispensable. Such studies will (i) clarify the impact of venom proteins on insects, if any, (ii) identify dose ranges that produce measurable developmental and reproductive defects, and (iii) reveal sub-lethal effects that could suppress pest population growth across generations. Addressing this gap will not only open new avenues for considering entomopathogenic nematode venom proteins in the discovery of novel insecticidal proteins but will also guide the rational design of integrated, environmentally sustainable pest management strategies.

## 2. Results

### 2.1. Venom Proteins Trigger Early-Instar Lethality and Prolong the Larval to Pupal Transition in Spodoptera frugiperda

**Figure 1:**
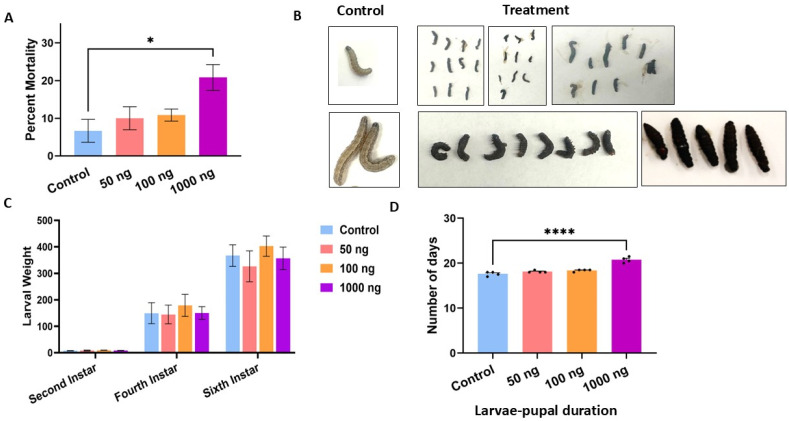
Effects of *S. carpocapsae* venom proteins on larval mortality, morphology, and development. (**A**) Percent mortality of the larvae fed on increasing concentrations of venom protein compared to the control. A significant increase in mortality was observed at the 1000 ng dose (* *p* < 0.05). (**B**) Representative images showing morphological differences between control and treated larvae. Control larvae appeared healthy and well-developed, whereas ~25% of the treated larvae exhibited signs of melanization, deformation, and arrested development in a dose-dependent manner. (**C**) Average larval weight across the second-, fourth-, and sixth-instar stages under different treatment conditions. Later stages showed reduced weight in treated larvae, but the difference was not significant. (**D**) The larval-to-pupal duration (in days) was found to be significantly prolonged in the treated groups compared to the control, with the most notable delay observed at the 1000 ng concentration (**** *p* < 0.0001).

In our solid diet feeding assay, *S. frugiperda* larvae displayed susceptibility to *S. carpocapsae* venom proteins. The average mortality rate reached approximately 22% at a concentration of 1000 ng g^−1^ diet, while cumulative mortality was around 12% for the control group (Figure 1A). The dietary doses, 50, 100, and 1000 ng ES protein g^−1^ diet, were deliberately kept within the physiological window estimated for ESP titers released during a natural *S. carpocapsae* infection (Supplementary Figure S1).

A significantly higher larval toxicity was observed in the case of early-instar larvae (Supplementary Figure S1). Although mortality also increased in late instars, the difference was not statistically significant. A fraction of larvae that ingested the highest dose, developed intense cuticular blackening before death (Figure 1B), indicative of systemic melanization. Surviving larvae across all treatment groups showed no significant deviation from control values in weight (Figure 1C), locomotor activity, food consumption, or frass consistency. However, individuals fed 1000 ng g^−1^ required a significantly extended larval-to-pupal interval (~1.5 days longer than controls; *p* < 0.0001), suggesting that the venom proteins impose a developmental cost even when toxicity is not lethal (Figure 1D).

### 2.2. Venom Proteins Compromise Pupal Viability and Reduce Adult Emergence

**Figure 2:**
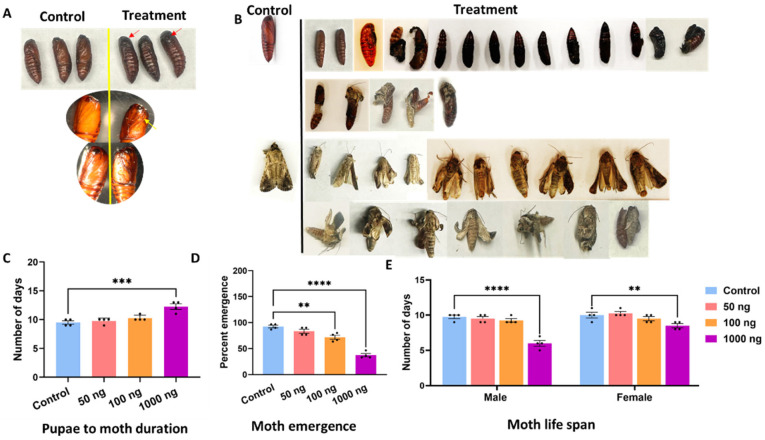
Oral administration of EPN venom proteins had deleterious impacts on pupal development, adult emergence, and moth lifespan. (**A**) Representative images of pupae from control and treated groups. Control pupae appeared normal in size and pigmentation. In contrast, treated pupae were smaller and darker with clear darker lines on the ventral surface (indicated with red arrow). (**B**) Phenotypic defects in pupae and adult moths from treated groups. Upper panels show abnormal pupae, represented by arrested melanized forms. Various abnormalities are illustrated including dehydrated, smaller, darker, and shrunken pupae. The extent of abnormality increased with increasing venom protein concentration and reached a maximum at the highest tested concentration (1000 ng). The middle and lower two panels depict defective moth emergence and a range of morphological deformities in the moths, respectively. The moth abnormalities include crumpled wings, incomplete eclosion, and deformed bodies in treated groups. (**C**) Duration (in days) from pupae to moth emergence increased significantly in treated groups compared to control, with the most pronounced delay observed at 1000 ng (*** *p* < 0.001). (**D**) Percent emergence of moths was significantly impaired in treated groups. There was a dose-dependent decline in successful emergence (** *p* < 0.01, **** *p* < 0.0001). (**E**) Moth lifespan was significantly reduced in both male and female moths, which emerged from venom protein-fed larvae. Male moths showed a longer delay (**** *p* < 0.0001) when compared with the females of the same group (** *p* < 0.01).

Ingestion of *S. carpocapsae* venom proteins during the larval stage led to dose-dependent defects in subsequent developmental stages (Figure 2). Pupae from treated larvae were darker, smaller, and morphologically distorted compared to controls (Figure 2A). Approximately 8.3% of treated larvae failed to pupate, and 12.4% of pupae and 13.3% of emerging adults were malformed. Consequently, only about 40% of treated larvae reached the adult stage compared to ~92% in controls.

Treated pupae that survived to adulthood required more time to do so. The pupal-to-adult interval extended by 0.9, 1.3, and 1.8 days at 50, 100, and 1000 ng, respectively (*p* < 0.001; Figure 2C). Adult emergence declined from 81% in controls to 44% at 100 ng and 19% at 1000 ng (*p* < 0.0001; Figure 2D). Median adult longevity also decreased significantly in both sexes (males: decreased from 10.4 ± 0.3 days to 7.1 ± 0.2 days; females: decreased from 10.6 ± 0.4 days to 8.3 ± 0.3 days; Figure 2E).

### 2.3. Venom Proteins Impair Reproductive Capacity and Egg Viability

**Figure 3:**
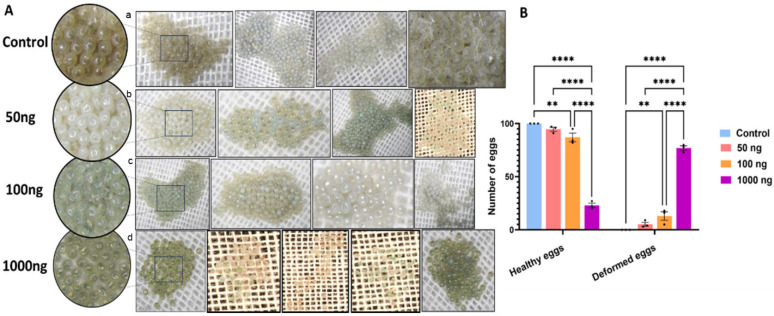
Oral administration of EPN ES significantly reduced fecundity and enhanced egg abnormality. (**A**) Images representing the egg-laying pattern of the moths, developed from the larvae that fed on a control diet (**a**) and 3 different concentrations of a venom protein mixed diet (**b**–**d**). The leftmost circular insets show a magnified view of the overall appearance of clustered eggs in various treatment groups. Panels (**b**–**d**) illustrate the morphological alterations in eggs and demonstrate a clear dose-dependent reduction in both the number of eggs laid and the rate of successful emergence. Notably, at higher concentrations (especially 1000 ng/gm of diet), eggs exhibit marked deformities and significantly decreased viability. (**B**) The bar graph represents the number of healthy (left bars) and deformed eggs per 100 eggs (right bars) across treatments and control. The number of healthy eggs was significantly reduced in the upper two treatment groups, in a dose-dependent manner. The data is presented as mean ± SEM. Statistical significance was assessed using ANOVA with post hoc multiple comparison tests: ** *p* < 0.01 and **** *p* < 0.0001.

Larvae that ingested *S. carpocapsae* venom proteins grew into adults with a striking, dose-dependent loss of reproductive capacity (Figure 3). Control moths laid dense, cream-colored egg clusters, whereas treated females produced smaller clusters with increasing numbers of flattened, translucent, and shrunken eggs (Figure 3A). The mean number of morphologically normal eggs per 100 fell by ~35% at 100 ng and by >90% at 1000 ng, while deformed eggs increased reciprocally (Figure 3B; *p* < 0.0001).

### 2.4. Venom Proteins Upregulate Antioxidative and Detoxification Systems in S. frugiperda

**Figure 4:**
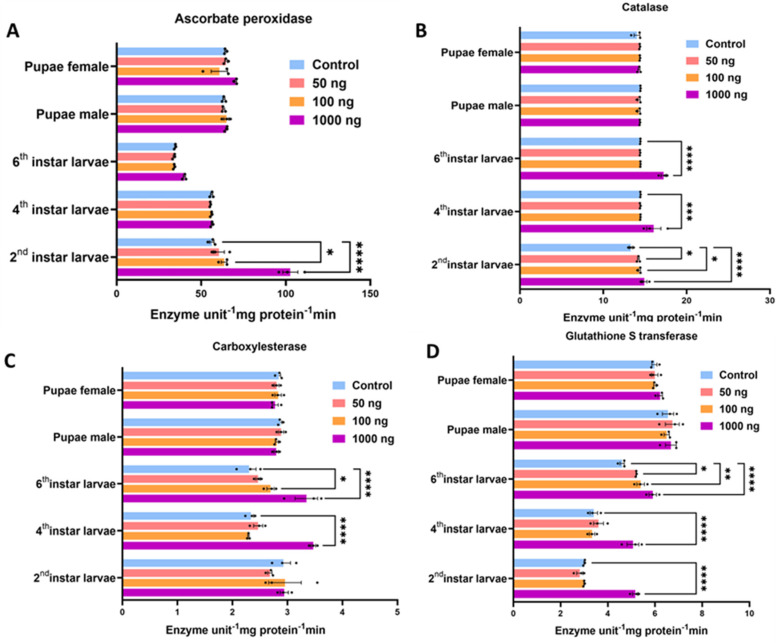
Treated larvae showed elevated enzyme activity for the antioxidant and detoxification enzymes across developmental stages. (**A**) Ascorbate peroxidase activity, a key antioxidant enzyme, notably increased in second-instar larvae in response to treatment, with a significant rise at 1000 ng (* *p* < 0.05, **** *p* < 0.0001). Activity in later larval stages and pupae remained relatively stable. (**B**) Catalase activity was significantly enhanced in all larval stages in a dose-dependent manner. The most pronounced effect was seen in second-instar larvae, suggesting an early-stage oxidative stress response (* *p* < 0.05, *** *p* < 0.001, **** *p*< 0.0001). (**C**) Carboxylesterase activity was measured in female and male pupae and second-, fourth-, and sixth-instar larvae. A dose-dependent increase in enzyme activity was observed and found to be particularly prominent in larval stages. The highest activity was noted at 1000 ng treatment, indicating an induced detoxification response (* *p* < 0.05, **** *p* < 0.0001). (**D**) Glutathione s-transferase (GST) activity shows a similar trend, with significantly elevated levels in all larval stages upon treatment, especially at the highest concentration. Female pupae exhibit only modest changes in GST activity compared to the control group (* *p* < 0.05, ** *p* < 0.01, **** *p* < 0.0001). Data are represented as mean ± SEM. Statistical significance was determined between the control and treatment groups for each developmental stage.

Dietary exposure to *S. carpocapsae* venom proteins significantly increased enzyme activity related to oxidative stress and detoxification pathways. Antioxidant enzymes such as ascorbate peroxidase (APX) and catalase (CAT) were elevated, particularly in early-instar larvae (Figure 4A,B). APX activity increased 2.5-fold in second-instar larvae fed 1000 ng (*p* < 0.05), and CAT activity was higher in all larval stages. Similarly, detoxifying enzymes, such as carboxylesterase (CE) and glutathione S-transferase (GST), showed significant upregulation. CE activity rose 2.3-fold over controls at 1000 ng g^−1^ in sixth-instar larvae (*p* < 0.001; Figure 4C), while GST activity nearly tripled in second-instar larvae (Figure 4D).

## 3. Discussion

The present study demonstrates that *Steinernema carpocapsae* venom proteins, when ingested through diet, can adversely affect the development and physiology of *Spodoptera frugiperda*. Unlike previous studies where either the venom proteins as a whole or the toxicity of individual components was demonstrated primarily through injection or hemocoelic exposure [4,6,18,20], our results show that dietary delivery alone can induce measurable larval mortality, growth inhibition, and delayed pupation. The concentrations tested (50, 100, and 1000 ng g^−1^ diet) were deliberately selected to fall within the physiological range estimated for *S. carpocapsae* venom protein titers released during a natural nematode infection, thereby reflecting ecologically realistic exposure levels [4]. We selected three distinct doses to capture the threshold effects of sub-lethal vs. lethal exposure. This focused design enabled us to capture the developmental and biochemical perturbations most representative of potential field-level exposure. Collectively, our findings indicate that at least a subset of the nematode’s venom proteins remains functionally active following ingestion, supporting their potential deployment as novel insecticidal candidates for crop protection.

The present findings show that *S. carpocapsae* venom proteins cause both lethal and sublethal effects in *S. frugiperda*. Early-instar larvae were particularly susceptible, consistent with previous reports that younger lepidopteran stages are generally more vulnerable to environmental and toxic stressors [21,22]. Comparable stage-specific susceptibility has also been observed with papaya and citrus seed powders [23,24] and with Bt-derived Vip3Aa proteins, which exhibit reduced efficacy in later instars [25].

The darker phenotype, observed at higher doses, is most likely the result of oxidative stress, cuticular darkening and/or melanization [26]. The available literature indicates that the interaction between *Steinernema* nematodes and the insect PO/PPO, melanization system is complex and context-dependent. In some host–nematode systems, live infection has been associated with increased PO activity, as reported for *S. carpocapsae* in the Colorado potato beetle [27] and for *S. feltiae* in *Helicoverpa armigera* [28]. In *Drosophila melanogaster*, *S. carpocapsae* infection was also associated with higher PO levels, particularly in response to axenic nematodes, suggesting that the host melanization response can be activated during nematode challenge [29]. On the other hand, studies of purified nematode-secreted molecules or cuticularly administered EPN venom proteins more commonly support suppression or interference with the melanization pathway, including a chymotrypsin-like protease, a trypsin-like protease, and a serpin that interferes with melanin incorporation into clots [11,14,17,30,31,32]. Taken together, the available literature and the cuticular darkening observed in our study suggest that modulation of the insect melanization cascade is context-dependent and influenced by multiple factors, including the nature of the bioactive molecules, the host system, and likely the route of delivery or exposure [30,33]. In our case, the observed darkening and associated mortality are best interpreted as part of a broader physiological response, most likely as an impact of multiple proteins present in venom (as we used total venom proteins rather than using a single purified protein), that may involve immune modulation, tissue damage, and oxidative stress, rather than a single defined mechanism. Possible contributors include exogenous proteases, which are highly represented in *S. carpocapsae* venom and may interact with pro-phenoloxidase-related pathways [33,34]; pore-forming toxins breaching midgut integrity [35]; or phospholipase-induced oxidative bursts [36]. All of these are represented in the *S. carpocapsae* secretome [4]. Given that over half of the secreted proteins are proteases or redox-active enzymes, the observed melanization and mortality likely stem from their synergistic action rather than a single toxic component.

Even in the absence of direct lethality, venom protein ingestion imposed developmental penalties, including delayed pupation, reduced pupal size, incomplete eclosion, and a shortened adult lifespan. Such delayed growth and reduced survival are hallmarks of nutritional or toxic stress during larval development and are known to translate into reduced fecundity and fertility in adult moths [37]. Similar outcomes have been documented for other biological insecticidal proteins, such as Cry toxins and protease inhibitors, highlighting that venom-derived proteins can act as potent oral toxins against lepidopteran pests [38,39].

The upregulation of antioxidative (CAT, APX) and detoxification (CE, GST) enzymes further supports the idea that the venom proteins are recognized as xenobiotic stressors by the insect’s physiology. These enzymatic responses are typical of insects encountering insecticidal compounds, whether synthetic (e.g., pyrethroids) or biological [40,41]. Persistent oxidative load, combined with impaired detox capacity, likely reflects systemic physiological imbalance associated with mortality, developmental arrest, and reproductive failure. It is important to note that these enzymatic changes serve as broad, correlative indicators of physiological stress following venom ingestion; the current data do not establish a direct mechanistic or causal pathway between these specific enzymes and the observed developmental disruptions.

Collectively, these results provide the first evidence that an EPN secretome can exert oral toxicity in a crop pest, impairing survival, development, and reproduction through both direct physiological disruption and indirect oxidative stress mechanisms, likely mediated by one or more proteins present in the venom.

## 4. Conclusions

Ingested *S. carpocapsae* venom proteins appear to target multiple aspects of *S. frugiperda* that begin in the mid-gut and echo through every later stage of *S. frugiperda* development. It starts most likely with the early-stage melanization-associated death in ~22% of the larvae. The protease-rich *S. carpocapsae* secretome is known to cleave host pro-phenoloxidase directly, causing the same melanization-associated response in caterpillars [4,42]. Protease action on pro-cuticle proteins can shrink and darken pupae [43]. Consistent with that report, ~60% of treated pupae failed to eclose or produced crumpled adults. We also recorded high activity of the oxidative stress enzymes in early-instar larvae, which was associated with delayed molting and an extended development period (larval–pupal development) following oral administration of the venom proteins. However, a direct causal link cannot be established based on the present data, though dietary stress is well known to shorten moth lifespan and egg output [40]. Treated adults lived 30% fewer days and laid up to 90% fewer normal eggs. These results are congruent with earlier work on plant protease inhibitors and Cry toxins [44,45]. Collectively, the results position *S. carpocapsae* venom proteins as promising candidates for next-generation biorational insecticides, capable of simultaneously curbing larval survival and adult reproductive success in a major lepidopteran pest. Future work should dissect the individual protein effectors and their applicability for the development of pest-resistant biotech crops.

## 5. Materials and Methods

### 5.1. Insects and Reagents

*Galleria mellonella* larvae were obtained from CritterGrub (Wausau, WI, USA), and *Spodoptera frugiperda* eggs were purchased from Benzon Research (Carlisle, PA, USA). A general-purpose lepidopteran diet (F9772) was obtained from Frontier Agricultural Sciences (Newark, DE, USA). All chemicals were of analytical grade. Phosphate-buffered saline (PBS), Triton X-100, potassium phosphate buffer, sodium dodecyl sulfate (SDS), α-naphthyl acetate, Fast Blue RR salt, α-chloro-2,4-dinitrobenzene (CDNB), reduced glutathione (GSH), hydrogen peroxide (H_2_O_2_), L-ascorbic acid, phenylmethylsulfonyl fluoride (PMSF), dithiothreitol (DTT), and protease inhibitor cocktail were purchased from Sigma-Aldrich (St. Louis, MO, USA). Protein concentrations were determined using the Qubit Protein Assay Kit (Thermo Fisher Scientific, Waltham, MA, USA).

### 5.2. Large-Scale Infection of Waxworm for the Collection of 24 Million Infective Juveniles of S. carpocapsae

*Steinernema carpocapsae* cultures were maintained at 16 °C in filtered tap water in cell culture flasks and used to infect waxworms (*Galleria mellonella*; CritterGrub). Approximately 2000 larvae were challenged at a dose of ~200 infective juveniles (IJs) per waxworm, following Lu et al. (2017) [4], with minor scale-up modifications. Briefly, 30 × 15 cm plastic containers were lined with filter paper moistened with filtered tap water containing ~2 IJs/µL, and ~80 waxworms were placed in each container. The containers were incubated at 25 °C for 1–2 days until larval death. Cadavers were then transferred to fresh containers lined with dry filter paper and allowed to dry for 5 days. Approximately 6–7 days post-infection, cadavers were rearranged in new containers, as shown in Supplementary Figure S2, to maximize IJ collection. Filtered tap water (10–12 mL) was added to the bottom of each container such that the corners of the filter paper contacted the water. From the following day through day 5, IJ-containing water was collected, and IJs were washed using a glass vacuum filter holder (Fisher Scientific, Pittsburgh, PA, USA) fitted with two 11 µm nylon net filters. The IJs were then stored in cell culture flasks at a concentration of 1–1.5 IJs/µL (200 mL per flask) and incubated at 16 °C for 3–6 weeks before being used for activation.

### 5.3. Activation of Infective Juveniles and Collection of Excretory-Secretory Proteins

For IJ activation, a 25% waxworm homogenate was prepared by grinding frozen waxworms in liquid nitrogen, followed by suspending the powder in PBS. The suspension was boiled 3–4 times in a microwave by gradual stirring and mixing, followed by placing it in a water bath to cool it down. After that, it was centrifuged at 3000 RCF for 5 min at 25 °C. The supernatant, including the lipid layer, was aliquoted, frozen, and supplemented with antibiotics immediately before use. Approximately 2 million *S. carpocapsae* infective juveniles (IJs) were washed multiple times in saline containing Triton X-100 and incubated at 25 °C in the dark on autoclaved sponge pieces soaked with waxworm homogenate and antibiotics to induce activation. After incubation for 6 h, IJs were gently recovered, extensively washed to remove contaminants, and resuspended in 50 mL 1X PBS and incubated on a shaker for 3 h at 150 RPM to allow continued venom release in liquid (PBS). After that, IJs were separated from the PBS containing venom proteins, using a glass vacuum filter holder fitted with two 11 µm nylon net filters. The protein preparation (excreted/secreted protein fraction) used here corresponds to the venom released by activated *S. carpocapsae* infective juveniles, as described in previous studies [4,6]. These venom proteins were filter-sterilized and concentrated using a 3 kDa Amicon Ultra centrifugal filter, quantified by a Qubit Protein Assay Kit, and stored at 4 °C. All activation and venom collection experiments were performed in multiple independent batches and pooled together.

### 5.4. Feeding Assay with Spodoptera frugiperda

*Spodoptera frugiperda* eggs were obtained from Benzon Research and held at 25 ± 2 °C until eclosion. Neonate larvae were used for the experiment. Larvae were maintained, and all the experiments were conducted at 25 ± 1 °C, 65 ± 5% RH, 16:8 h L:D. A general-purpose lepidopteran diet was prepared according to the manufacturer’s instructions and supplemented with venom proteins at 50, 100, or 1000 ng g^−1^ diet, individually after dilution into 1 mL of PBS. For this, equal amounts of diet were weighed into four different Petri plates, crushed using a mortar and pestle, and thoroughly mixed with the respective amounts of venom protein/control PBS, using a spatula. The selected dietary doses were deliberately kept within the physiological window estimated for ESP titers released during a natural *S. carpocapsae* infection [4]. A diet containing an equal volume of PBS served as the control. Newly hatched larvae were placed individually in sterile, ventilated glass tubes containing 2 g of the appropriate test diet. Each treatment and the control comprised 30 larvae, and the entire experiment was repeated four times. Food was replaced with a fresh, identically treated aliquot every second day, beginning on day 6, and continued until pupation. Larval mortality, weight gain, and diet consumed were recorded every 48 h throughout the larval duration. Larvae failing to respond to gentle prodding were recorded as dead and removed. A high-precision semi-analytical balance (OHAUS Adventurer^®^ Balance Scale) was used for the weight measurements of larvae and unconsumed food diet. Pupae remained in their original tubes under the same environmental conditions. Moisture was maintained by adding a few drops of distilled water daily, and adult emergence was recorded. Sex was determined on day 2 of the pupal stage, once the cuticle had hardened [46]. Because sexing is possible only at the pupal stage, each larva was tracked individually throughout development. Data were recorded for the number of adults that emerged per treatment and phenotypic abnormalities associated, if any.

### 5.5. Fecundity Assessment

To examine reproductive behavior, 1- to 2-day-old males were paired with age-matched females from the same treatment group. Three pairs per dose (50, 100, and 1000 ng g^−1^ diet) and three control pairs were each placed in separate plastic beakers. Every beaker contained a cotton-plugged Petri dish holding a liquid diet (5% sucrose + 5% honey). The inner wall of the beakers was lined with white A4 paper, and the mouth was covered with muslin cloth to provide an oviposition substrate and ventilation. For each pair, we recorded the spatial egg laying pattern, number of eggs per unit area, and egg morphology, including external traits such as size, shape, and color.

### 5.6. Protein Isolation from Larvae and Pupae for Enzyme Assay

Second-, fourth-, and fifth-instar larvae of comparable size and weight, together with male and female pupae from each treatment and the control, were processed individually for protein extraction. After a brief rinse in distilled water, specimens were immobilized on ice for 30 min, snap-frozen in liquid N_2_, and ground to a fine powder with a pre-chilled mortar and pestle. The powder was resuspended in ice-cold 1 × PBS supplemented with 1 mM PMSF, 1 mM DTT, and a 1 × protease inhibitor cocktail, using a tissue-to-buffer ratio of 1:8 (*w*:*v*). Total protein was quantified with the Qubit Protein Assay, and working solutions were standardized to 100 µg mL^−1^ in 1 × PBS for downstream enzyme assays.

### 5.7. Measurement of the Detoxifying and Antioxidant Enzyme Activities

Protein extracts standardized to 100 µg mL^−1^ were assayed for key enzymes from the two functional groups most often perturbed by insecticides: detoxification and antioxidative defense. Carboxylesterase and glutathione S-transferase were quantified as detoxification markers, while catalase and ascorbate peroxidase represented the antioxidative enzymes. For all the enzymatic reactions, absorbance was recorded on SpectraMAX iD3 multi-mode microplate reader (Molecular Devices, San Jose, CA, USA) using a microplate reader. Enzyme activity was calculated from the slope of the linear portion of the reaction curve, using their respective extinction coefficients (ε), and expressed as enzyme units (µmol product formed) per milligram protein per minute (U mg^−1^ min^−1^). Blanks containing PBS were run in parallel to correct for non-enzymatic hydrolysis, and all samples were assayed in 3 biological replicates [47].

#### 5.7.1. Carboxylesterase Enzyme Assay

For the carboxylesterase assay, 50 µL of protein extract (100 µg mL^−1^) was combined with 1.35 mL of 0.27 mM α-naphthyl acetate in a 2 mL VWR microcentrifuge tube and incubated at 30 °C for 25 min. The reaction was terminated with 50 µL of Fast Blue RR salt (1 mg mL^−1^ in PBS) followed by 10 µL of 5% (*w*/*v*) sodium dodecyl sulfate. Tubes were left at room temperature for color development (15–20 min), absorbance was recorded at 600 nm, and the enzyme activity was calculated from the linear portion of the reaction curve, using an extinction coefficient (ε) of 2.2 mM^−1^ cm^−1^.

#### 5.7.2. Glutathione-S-Transferase (GST) Assay

GST activity was quantified following the CDNB conjugation method with minor modifications [47]. Briefly, α-chlorodinitrobenzene (CDNB) and reduced glutathione (GSH) were prepared as 10 mM stocks in ethanol and 1 × PBS, respectively. For each reaction, 100 µL of clarified larval homogenate (100 µg protein mL^−1^) was combined with 100 µL CDNB (final 1 mM) and 100 µL GSH (final 1 mM) in a 1.5 mL VWR microcentrifuge tube; the volume was adjusted to 1 mL with ice cold 1 × PBS (pH 7.4). The increase in absorbance at 340 nm was recorded every 30 s for 5 min at 25 °C. Enzyme activity was calculated from the linear portion of the curve with an extinction coefficient (ε) of 9.6 mM^−1^ cm^−1^.

#### 5.7.3. Catalase (CAT) Assay

Catalase activity was determined by monitoring the decomposition of H_2_O_2_, following Aebi (1984) with minor adaptations [48]. Each 1 mL reaction contained 50 µL of larval homogenate (100 µg protein mL^−1^) and 950 µL of 50 mM potassium phosphate (KPi) buffer, pH 7.0. The reaction was initiated by adding 10 µL of freshly prepared 1% (*w*/*v*) H_2_O_2_ (final concentration ~3 mM). The decline in absorbance at 240 nm that corresponds to H_2_O_2_ decomposition was recorded every 10 s for 5 min at 25 °C. The extinction coefficient (ε) of 39.4 mM^−1^ cm^−1^ was used to calculate the enzyme activity.

#### 5.7.4. Ascorbate Peroxidase (APX) Assay

APX activity was quantified by monitoring the H_2_O_2_-dependent oxidation of ascorbic acid as described by Nakano and Asada (1981) with slight modifications [49]. The reaction mixture (1 mL total volume) contained 100 µL clarified larval homogenate (100 µg protein mL^−1^), 250 µL 200 mM potassium phosphate buffer (KPi), pH 7.0, 300 µL 2 mM L-ascorbic acid (final concentration 0.6 mM), and 340 µL deionized water. Then, 190 µL of the reaction mixture was aliquoted into a 96-well plate in quadruplicate. The reaction was initiated by adding 10 µL of freshly prepared 10% (*w*/*v*) H_2_O_2_ (final concentration ~3 mM). The decrease in absorbance at 290 nm, corresponding to ascorbate oxidation, was recorded every 30 s for 3 min. Enzyme activity was calculated from the initial linear rate of absorbance change, employing an extinction coefficient (ε) of 2.8 mM^−1^ cm^−1^.

### 5.8. Statistical Analysis

All statistical tests were carried out in GraphPad Prism 10.2.2. For larval weight data, the two highest and two lowest values in each treatment and the control were excluded as outliers before analysis. Mean larval weight, numbers of pupae and adults, and specific activities of carboxylesterase, GST, catalase, and APX were analyzed by one-way ANOVA. When the omnibus F test was significant, treatment means were compared with the control by Dunnett’s multiple comparison test. Data are presented as mean ± SEM, and differences were considered significant at *p* < 0.05.

## 6. Patents

The results described in this work are related to the patent application WO2025265062A1, titled “Nematode toxins and methods of use,” filed by the University of California, on which M.M. and A.R.D. are listed as inventors.

## Data Availability

The original data presented in the study are openly available in FigShare at 10.6084/m9.figshare.31395831.

## Supplementary Materials

The following supporting information can be downloaded at: https://www.mdpi.com/article/10.3390/toxins18040185/s1, Figure S1: Venom protein diet shifts the developmental progression of *Spodoptera frugiperda*. (A) Stacked-bar plot showing the percentage of individuals (mean ± SEM, *n* = 4 replicates of 30 larvae) that reached each developmental category after feeding on control diet or diet containing 50, 100, or 1000 ng g^−1^ *S. carpocapsae* venom proteins. Categories are ordered chronologically from bottom (early-instar death) to top (emerged female moths). Asterisks mark significant differences from the control within each category (one-way ANOVA + Dunnett; ** *p* < 0.01, **** *p* < 0.0001). (B) Schematic summary: the green arrow indicates increasing early-stage mortality and developmental arrest with rising venom protein dose, whereas the red arrow denotes the concomitant decline in normal adult output; Figure S2: Schematic representation of the method used for waxworm infection and IJ activation.

## Abbreviations

The following abbreviations are used in this manuscript:

APX	Ascorbate Peroxidase
CAT	Catalase
CE	Carboxylesterase
EPN	Entomopathogenic Nematodes
GST	Glutathione S-Transferase
