# Supplementary material for: Entomopathogenic Nematode Steinernema carpocapsae Venom Proteins Disrupt Developmental Physiology and Reproduction of Spodoptera frugiperda (Lepidoptera: Noctuidae)
Source: Toxins (Basel). 2026 Apr 14;18(4):185. doi: 10.3390/toxins18040185 (PMC13119690; doi:10.3390/toxins18040185)
Supplement: Supplementary file 1 [file toxins-18-00185-s001.zip › toxins-4209735-supplementary.pdf]

Supplemental Materials: Entomopathogenic nematode *Steinernema carpocapsae* venom proteins disrupt developmental physiology and reproduction of *Spodoptera frugiperda* (Lepidoptera: Noctuidae)

Manisha Mishra, Leonor Georgette Farias, Steven Song, Steven Nguyen, Purav Shah, and Adler R Dillman

EPN venom proteins

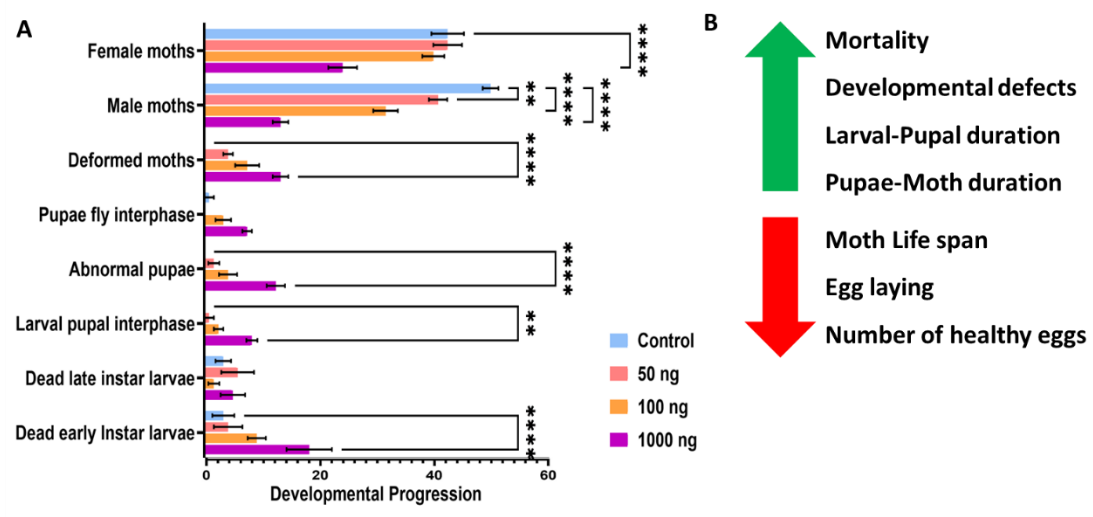

Figure S1. Venom protein diet shifts the developmental progression of *Spodoptera frugiperda*. **(A)** Stacked-bar plot showing the percentage of individuals (mean ± SEM, n = 4 replicates of 30 larvae) that reached each developmental category after feeding on control diet or diet containing 50, 100, or 1000 ng g^{-1} *S. carpocapsae* venom proteins. Categories are ordered chronologically from bottom (early-instar death) to top (emerged female moths). Asterisks mark significant differences from the control within each category (one-way ANOVA + Dunnett; ** $p < 0.01$, **** $p < 0.0001$). **(B)** Schematic summary: the green arrow indicates increasing early-stage mortality and developmental arrest with rising venom protein dose, whereas the red arrow denotes the concomitant decline in normal adult output.

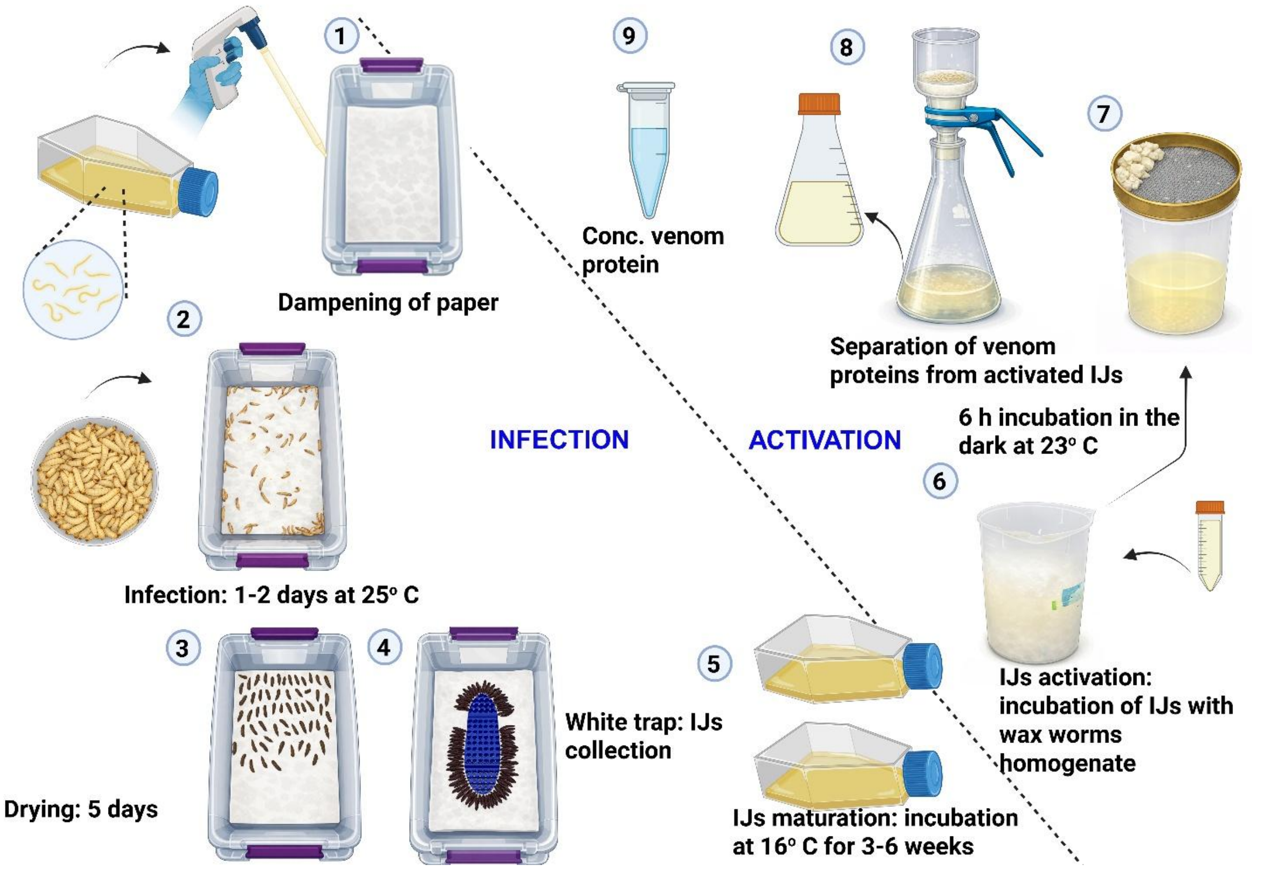

Figure S2. Schematic representation of the method used for waxworm infection and IJ activation.
